# Modeling of the Arabian/Nubian shield’s geothermal structure: a multi-parametric analysis using geophysical and geological tools

**DOI:** 10.1038/s41598-025-33846-2

**Published:** 2026-01-12

**Authors:** Menna Haggag, Mohamed Sobh, Soha Hassan, Mahmoud Abd El-Rahman Hegab, Ali Shebl, Hosni H. Ghazala

**Affiliations:** 1 Geology Department, Faculty of Science, Mansoura University, Mansoura, 35116 Egypt; 2 Leibniz Institute for Applied Geophysics (LIAG), 30655 Hannover, Germany; 3 Geodynamics Department, National Research Institute of Astronomy and Geophysics (NRIAG), Helwan, 11421 Cairo Egypt; 4 Mineral Resources Department, National Authority for Remote Sensing and Space Sciences, Cairo, Egypt; 5 Department of Geology, Tanta University, Tanta, 31527 Egypt; 6 Department of Mineralogy and Geology, Faculty of Science and Technology, University of Debrecen, Egyetem tér 1, Debrecen, 4032 Hungary

**Keywords:** Arabian–Nubian shield, Geothermal structure, S-wave tomography, Lithospheric structure, Heat flow, Mantle upwelling, Climate sciences, Solid Earth sciences

## Abstract

The Arabian-Nubian Shield (ANS) is a complex Neoproterozoic tectonic mosaic whose lithospheric structure and geothermal regime remain poorly constrained. Here, for the first time across the entire ANS, we integrate S-wave tomography, seismic velocity models, lithospheric density from Gravity Field and Steady-State Ocean Circulation Explorer (GOCE) satellite data, and Land Surface Temperature (LST) derived from Moderate Resolution Imaging Spectroradiometer (MODIS) imagery to map crustal and upper mantle architecture. Results show stark lithospheric contrasts: the stable Arabian Platform has thick, cold lithosphere (Moho depth ~ 32.5 km, lithosphere-asthenosphere boundary (LAB) > 200 km) and low heat flow (40–50 mW/m²), whereas the Arabian Shield and Red Sea rift exhibit thin lithosphere (Moho 25–30 km, LAB 60–120 km) with high heat flow (70–90 mW/m²). These areas display low S-wave velocities (≤ 3920 m/s), reduced mantle density, and high-temperature anomalies indicative of active mantle upwelling and lithospheric thinning. This multi-parametric framework identifies zones of pronounced lithospheric thinning as prime targets for geothermal exploration across the ANS and provides new insight into its Cenozoic geodynamic evolution.

## Introduction

**Fig. 1:**
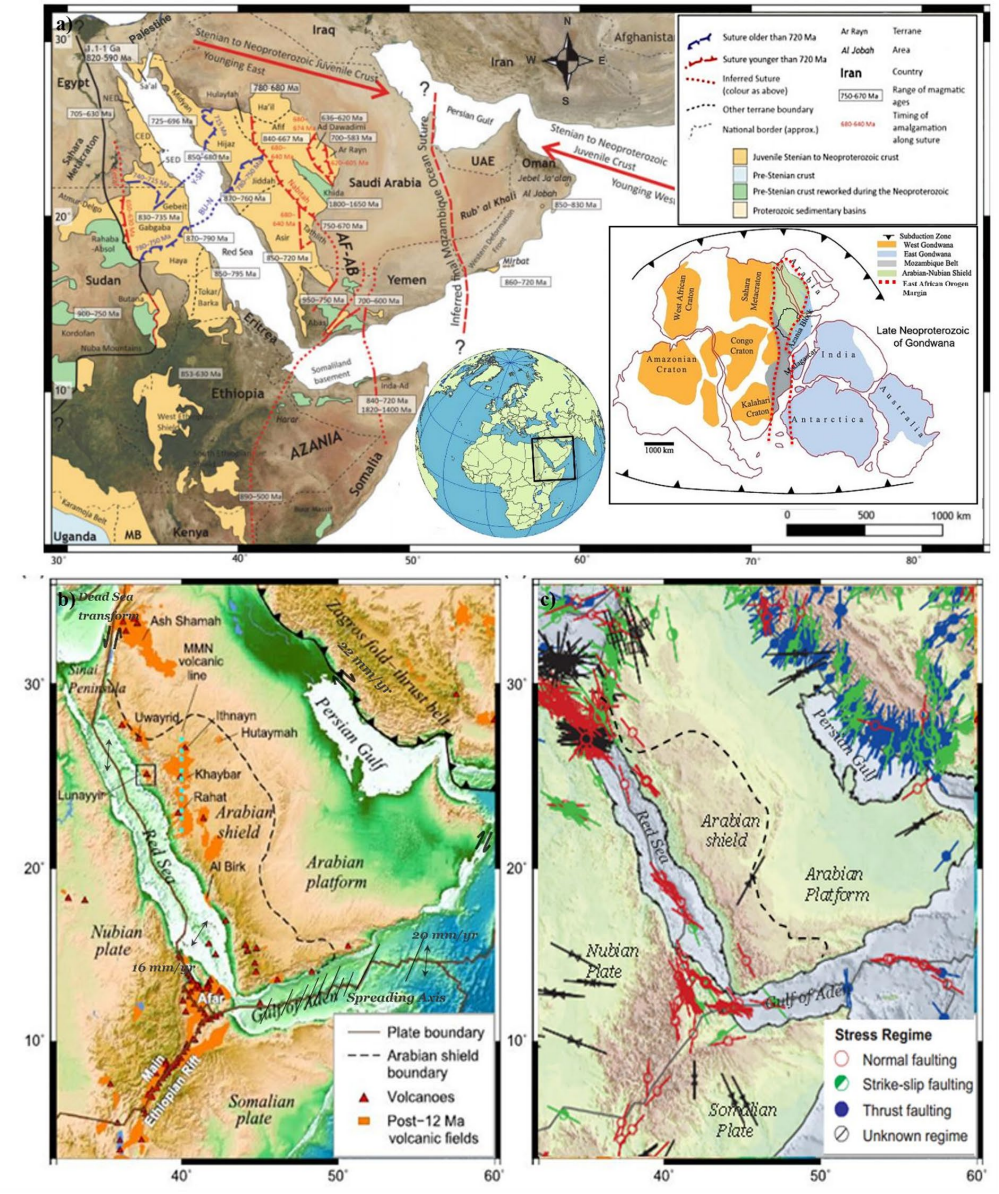
Modified northern Indian Ocean crust age map, showing pre-Stenian and Stenian-Ediacaran crust^20^ (**a**), Study area map with plate boundaries, volcanoes, and post-12 Ma volcanic fields^21^(**b**), Stress map of the study area^35^(**c**).

 The Arabian/Nubian Shield (ANS) is a critical geological region spanning northeastern Africa and the Arabian Peninsula, including Saudi Arabia, Jordan, Palestine, Yemen, Egypt, Sudan, Kenya, Ethiopia, and Eritrea (Fig. 1a). It was formed during the Neoproterozoic Era (ca. 870–530 million years ago)^1^. The ANS showcases intraoceanic island arcs, granitoid intrusions, and volcano-sedimentary sequences, marking a significant episode of crustal growth during the transition from the fragmentation of the Rodinia supercontinent to the assembly of Gondwana^2^. Its geological mosaic comprises distinct tectonic terranes, shaped by collisions and accretion over millions of years^1^. These terranes are primarily composed of granitoids, metavolcanics, and metasedimentary rocks, often separated by suture zones and remnants of ancient ocean basins^3^. The study of these terranes offers insights into the processes of island arc formation, subduction, and continental collision that shaped the ANS^4^.

Approximately 30 million years ago, the Arabian Plate began separating from the African Plate, triggering rifting along the Red Sea and the Gulf of Aden^5,6^. This process led to the formation of extensive flood basalts in northern Ethiopia and Yemen, known as the Ethiopia-Yemen traps^7^. The origin of the Red Sea remains debated, with hypotheses suggesting passive rifting driven by slab pull at the Zagros subduction zone or active rifting caused by upwelling mantle plumes^8,9^. Western Arabia’s volcanic activity during the Cenozoic is also a topic of ongoing discussion. The region’s Cenozoic volcanic fields, or “harrats,” are categorized into older basalts (30–20 million years old) that are tholeiitic to transitional in composition and younger basalts (12 million years to present) that are transitional to strongly alkalic^10^ (Fig. 1b).

The ANS’s geological evolution is marked by a complex interplay of stress regimes and tectonic processes. During the Neoproterozoic Era, significant compression led to the formation of thrust faults and fold belts, driven by tectonic terrane collisions^11^. The breakup of Gondwana in the Mesozoic and Cenozoic introduced extensional forces, resulting in normal faults and rift basins^12^. Prominent fault systems, such as the Najd Fault System, the Red Sea Rift, and the East African Rift System, highlight the region’s dynamic tectonic history^13^. Later-stage strike-slip motion in certain areas reflects changes in stress orientation^14^. These fault systems influence fluid circulation, permeability, and heat distribution, directly impacting the region’s geothermal heat flow potential^15^ (Fig. 1c).

Geothermal heat flow in the ANS is shaped by both radiogenic heat production within the crust and heat transfer from mantle upwelling^16^. The extensional regimes associated with rifting enhance mantle-derived heat contributions to the crust^17^. Faults and fractures act as conduits for hydrothermal fluids, while suture zones and shear zones control fluid movement^18^. Interactions between hydrothermal fluids and surrounding rocks result in mineral deposits and alteration zones, which serve as critical indicators of geothermal activity^19^.

Beyond its geological significance, the ANS holds substantial economic value due to its mineral resources, including gold and copper^20^. It also serves as a natural laboratory for studying the formation of continental crust and early tectonic processes. However, the ANS remains understudied from a geophysical perspective, with most research focusing on specific sections, limiting a comprehensive understanding of its characteristics^21^. To address this, our study integrated geophysical and geological data to explore the geothermal potential of the Arabian Shield. By interpreting satellite gravity and magnetic projects data, we estimated the Moho and Lithosphere-Asthenosphere Boundary (LAB) to investigate the region’s upper mantle structures. Additionally, we developed geothermal heat flow models to predict subsurface heat distribution and identify high-potential areas for sustainable energy^22^. Numerous studies have been conducted to explore the geological and geothermal characteristics of the ANS, highlighting its complex structure and geothermal potential^23–30^. These investigations are broadly categorized into two main areas: geology-stratigraphy and geothermal studies (Table 1). About geology-stratigraphy, several studies have investigated the geological structure and stratigraphy of the ANS, revealing significant variations in crustal and mantle properties. Celli et al. (2020)^31^ supposed a thick cratonic lithosphere beneath the Arabian Platform that suggested unexposed Archean crust. The craton boundary near the Oman Mountains defines the boundary between the Persian Gulf and Gulf of Oman and separates the Zagros and Makran subduction zones^32^. Harash et al. (2023)^23^ reported Moho depths exceeding 40 km in the western Arabian Shield and thinning to 16–20 km beneath the Mediterranean Sea.

Al-Amri et al. (2008)^24^ identified lower velocity zones and Moho depths ranging from 22 to 40 km, suggesting higher temperatures. Kaban et al. (2016) and Benoit et al. (2003)^25,26^ highlighted mantle density asymmetries and evidence of upwelling affecting the region’s structure. Förster et al. (2009) and Rolandone et al. (2013)^27,28^ estimated lithospheric thickness at around 150 km, indicating significant thinning and mantle dynamics. As for geothermal studies, they have revealed key aspects of the thermal regime of the ANS. Salem et al. (2024)^29^ noted an average surface heat flow of 50 mW/m², influenced by radiogenic heat and mantle dynamics. Rolandone et al. (2013)^28^ reported regional variations, ranging from 45 mW/m² in the eastern Shield to 60 mW/m² in the west. Förster et al. (2009) and Park et al. (2007)^27–30^ linked thermal anomalies to mantle upwelling and lithospheric thinning, with heat flow averaging 24–29 mW/m². Al-Amri et al. (2008)^24^ connected elevated geothermal gradients to Cenozoic volcanic activity and tectonic uplift. An alternative approach capitalizes on the well-established correlation between seismic mantle velocities and temperature identified the LAB as typically situated within the temperature range of 1250 to 1350 °C^33,34^.

This research aims to develop a high-resolution geothermal heat flow (GHF) model for the Arabian-Nubian Shield region. Given the limited availability of direct temperature gradient data from boreholes, the study addresses the challenges of assessing geothermal resources in this vast and geologically complex area. To overcome these constraints, we integrate, for the first time across the entire ANS, multiple satellite-derived datasets alongside on-the-ground geophysical and geological data. The novelty of this work lies in its continental-scale, multi-parametric integration of S-wave tomography, GOCE gravity data, magnetic data for Curie Point Depth, and MODIS thermal data to produce a unified lithospheric and geothermal model.

**Table 1:** Previous studies have been conducted that explore the geological and geothermal characteristics of the ANS.

Study	Region	Insights	Datasets	Results
^23^	Eastern Mediterranean Sea	Primarily focuses on Moho depth variations and their implications for geodynamics and seismic activity in the region.	• Bouguer gravity data Seismic results for validation of inverted findings.	• Maximum Moho depth exceeds 40 km in western Arabian shield. Minimum Moho depth of 16–20 km beneath Mediterranean Sea.
^24^	the Arabian Nubian Shield and Red Sea	• Lower velocity zones indicating higher temperatures.	• 3-component broadband velocity seismograms for Mw > 5.8 earthquakes. • High-quality waveform data from KACST and SGS.	• Moho depths vary from 22–25 km near the coast to 35–40 km inland. • The LAB depth varies from 55 km near the coast to 100–110 km beneath the Shield.
^25^	The Arabian Shield	• The Arabian Shield exhibits dense upper mantle material, with thermal variations linked to seismic velocities. Density variations reveal asymmetry in Arabian plate structure.	• Seismic data from tomography and crustal models. • Gravity and topography data for inversion analysis.	• A three-dimensional density model variation, reaching up to ± 60 kg/m³, • East African Rift decreases in mantle density, the northern Red Sea is about 150 km depth, • The Arabian Shield has a high-density uppermost mantle due to past mantle upwelling, while the Arabian Platform’s lower part of the upper mantle is relatively dense
^26^	The Arabian Shield	The Arabian Shield exhibits significant thermal variations, with a broad low-velocity anomaly in the upper mantle.	• 293 station-event pairs from Saudi Arabia PASSCAL Experiment. • Broadband seismic data from the Saudi Arabian PASSCAL Experiment.	• Transition zones vary from 250 to 275 km, • The low velocity anomaly is confined to depths shallower than 410 km
^27^	The Arabian Shield and Red Sea	• The Arabian Shield exhibits significant thermal variations due to mantle upwelling and lithosphere thinning.	• Sample Collection, • Chemical Composition, Petrophysical Properties” Density, Thermal Conductivity, Radiogenic Heat Production” • Previous Surface Heat Flow Data	• LAB depths about (110–160 km) • Moho depths between 35–40 km • Moho temperatures range from 530–650 °C • Geothermal heat flow, averages between 24 and 29 mW m^− 2^.
^28^	Oman and the Arabian Shield and Platform		• Data Collection from Boreholes • Geological Context	• The average surface heat flux is 45 mWm − 2 across the eastern Arabian Shield, low crustal heat production values of 0.7 µWm − 3 • The eastern region heat flow of 45 mWm − 2, while the western part around 60 mWm − 2 • The lithospheric thickness for the Arabian plate is approximately 150 km
^29^	The Arabian Shield	• Curie depth varies across Arabian Plate, deepest at center. • Surface heat flow increases towards west and east of the plate. • Variations in the upper mantle elevation and crustal radiogenic heat contribute to geothermal heat flow differences across the region.	• Aeromagnetic data for spectral analysis. • Shallow temperature measurements for geothermal surveys.	• The Arabian Shield exhibits low bulk radiogenic heat production (0.4 µW/m^3^) • a surface heat flow of around 50 mW/m^2^.
^30^	The Arabian Peninsula and northern Red Sea	• Attributed these low velocities to a thermal anomaly in the upper mantle, linked to the Cenozoic uplift and volcanic activity.	• SANDSN data from twenty-two broadband and eleven short-period seismographs. • Data from permanent stations RAYN, EIL, and MRNI.	• Low velocity indicates temperatures up to 330 K.

### Methodology and datasets

To accurately investigate the geothermal characteristics of the Arabian Shield, our approach focuses on developing a comprehensive model of the crustal and lithospheric structure. This model integrated key geophysical properties such as density, seismic velocity, and thermal conductivity of the upper mantle. Serving as the cornerstone of our geothermal heat flow analysis, the model enabled precise calculations of heat transfer within the region. The methodology began by determining the crustal thickness, which was established as a fixed parameter. This provided a stable framework for examining the thermal structure and dynamics of the upper mantle. This provides a stable framework for examining the thermal structure and dynamics of the upper mantle.

To ensure high-resolution results, our research methodology intentionally filters out peripheral contributions, allowing us to concentrate on precisely mapping the Moho and LAB structural characteristics. The methodology employs a systematic approach to develop geothermal heat flow models for the study region. This process began with the acquisition of geological and geophysical datasets, including satellite observations, seismic constraints, and findings from previous studies, with an emphasis on factors such as geological formations and temperature gradients. The subsequent data processing phase involved applying essential corrections to the gravity data to isolate the anomalous density distribution associated with Moho depth variations. The refined gravity data were then utilized in the inversion process, where probabilistic methods generated subsurface realizations based on prior probability distributions to evaluate the effect of uncertainty on lithologic and density structures. This was followed by preliminary modeling, which integrated the joint inversion of geophysical and geological data to model temperature, heat flow, and geothermal gradients. To ensure robustness in data-scarce regions, temperature gradients were estimated using correlational methods, machine learning, and expert collaboration to address data gaps. Finally, the models were iteratively refined and validated against independent datasets.

### Datasets

The dataset used in our study encompasses multiple geophysical and geological datasets. Specifically, the study implemented the following types of data: (a) Satellite-derived Gravity Data: high-resolution gravity data obtained from the GOCE (Gravity field and Ocean Circulation Explorer) satellite, (b) Magnetic Data: The magnetic data used in this study were compiled from the World Digital Magnetic Anomaly Map (WDMAM) and supplemented with regional aeromagnetic surveys. This dataset provides a high-resolution map of the magnetic field anomalies across the Arabian-Nubian Shield, which is essential for estimating the Curie Point Depth (CPD), a key thermal parameter., (c) global Seismic velocity Data: Data obtained from seismic experiments to infer crustal and thermal structures and the recent S wave tomographic model of Africa AF2019 provided by^31^, (d) Geological Data: that related to regional geological formations, including temperature gradients, Surface mapping data that helps in constructing the geological model, and (e) Topographic Data: global topography data from sources such as ETOPO1 for essential corrections related to gravity data^23–34,36–38^.

In this study, the raw gravity data were derived from the satellite-only spherical harmonic model GOCO6s^39^, The satellite gravity data utilized in this study is derived from the GOCO6S model, which is the latest release of the Gravity Observation Combination (GOCO) project initiated under the European Space Agency’s (ESA’s) Gravity Field and Steady-State Ocean Circulation Explorer (GOCE) mission. Launched in 2009, GOCE was designed to determine the Earth’s gravity field and geoid with high precision and a spatial resolution of approximately 80 km^40^. The GOCO6S model provides a satellite-only global gravity field model up to degree and order 300, with constrained secular and annual variations up to degree and order 200, achieving a spatial resolution of 70 km. This high-resolution and high-accuracy in the medium-to-long wavelength range makes the GOCO6S model particularly suitable for resolving the deep-seated density structures of the lithosphere and upper mantle, such as the Moho and LAB, which are the primary focus of our regional-scale study. The Bouguer anomaly (BA) signal was synthesized at a constant altitude of 50 km above the ellipsoid, a height chosen to maintain signal clarity while minimizing noise amplification^41^. These synthesized BA data served as the basis for estimating the initial depths of the Moho and the LAB. The use of GOCO06s is motivated by its enhanced processing chain, high accuracy, and ability to resolve long-wavelength gravity anomalies associated with deep-seated crustal and mantle structures. The dataset is accessible through the International Centre for Global Earth Models (ICGEM) service (https://icgem.gfzpotsdam.de/ICGEM/)^42^.

In addition to geophysical datasets, this study employed Moderate Resolution Imaging Spectroradiometer (MODIS) Land Surface Temperature (LST) data to assess surface thermal patterns and identify potential geothermal anomalies across the ANS. The analysis was performed using Google Earth Engine (GEE). The primary dataset used was the MODIS/Terra Land Surface Temperature and Emissivity Daily L3 Global 1 km SIN Grid (MOD11A1), which provides daily LST observations at a spatial resolution of 1 km. The dataset was spatially constrained to the study area and temporally filtered to include observations from January 1, 2016, to December 31, 2023. The LST_Day_1km band was selected, and a scale factor of 0.02 was applied to convert the digital values into physical units (Kelvin). The overarching objective of this approach was to evaluate whether surface thermal characteristics derived from satellite observations are consistent with subsurface conditions inferred from geophysical data.

## Data processing

The magnetic data were processed using spectral analysis (specifically, the centroid method) to estimate the Curie Point Depth (CPD). The data were first corrected for the International Geomagnetic Reference Field (IGRF) and then upward-continued to a common datum to minimize near-surface noise and enhance the deeper crustal signals. The centroid method involves calculating the power spectrum of the magnetic anomalies in the wavenumber domain, where the slope of the deeper portion of the spectrum is used to determine the depth to the bottom of the magnetic source (the Curie isotherm).

**Fig. 2:**
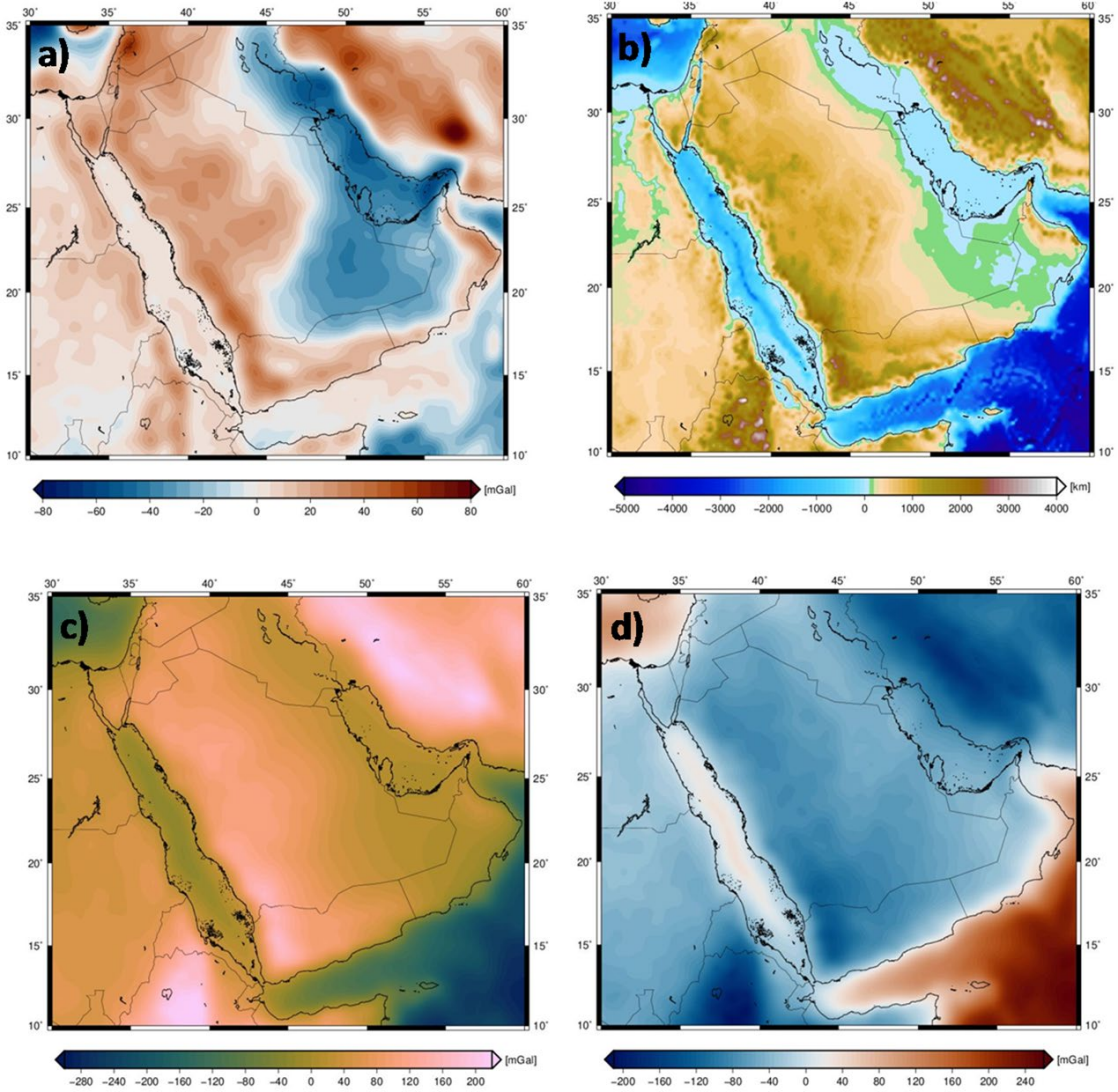
Gravity disturbance from GOCO6S satellite model **(a**), Topography from ETOPO1(**b**), Gravitational attraction of the topography calculated at the observation height using tesseroids (**c**), Bouguer anomaly obtained by subtracting the gravitational effects of the topography from gravity disturbance (**d**).

In the gravity field modeling, isolating the anomalous density distribution is essential before inversion. This study focuses on characterizing the undulating Moho relief relative to Moho, necessitating the removal of all other gravity contributions from the observations. Gravity disturbances were calculated by subtracting the scalar gravity of the ellipsoidal reference Earth, as determined using the closed-form solution of^43^. These disturbances retained anomalous signals associated with terrain, sediment, and elevation variations (Fig. 2a).

To account for topographic influences, the topographical correction (TC) signal was estimated using the tesseroids software program^44^. This method is highly accurate as it models the terrain using three-dimensional spherical prisms (tesseroids). The correction was computed using the ETOPO1 global topography model [25] with a resolution of 1 arc-minute (Fig. 2b). To ensure the accuracy of the regional model and to reduce border effects, the calculation was extended with a 5-degree padding beyond the study area in all directions^45^ (Fig. 2c). A uniform density of 2670 kg/m³ was assumed for the upper continental crust and 1030 kg/m³ for offshore areas, as detailed topographic density data was unavailable. This value was selected after several trials confirmed that it produced the optimal model performance, providing the best fit to independent seismic constraints. This meticulous processing refined the gravitational data to isolate the signal from subsurface density variations, ensuring the final Bouguer disturbance was free from the dominant influence of topography. A sensitivity analysis confirmed that while the absolute Moho depth is influenced by this parameter, the overall spatial pattern of Moho topography—most importantly, the fundamental contrast between the shallow Arabian Shield and the deep Arabian Platform—is robust. This confirms that our primary tectonic interpretations are not an artifact of the specific density value chosen. A Bouguer anomaly was obtained by subtracting the gravitational effects of the topography from the gravity disturbance (Fig. 2d). This process is crucial in isolating the gravitational signal caused by subsurface density variations^46^. By removing the topographic effects, the corrected gravity signal reflects more accurately the subsurface features, such as the Moho and the LAB^47^. The Bouguer correction is typically applied after considering the terrain, bathymetric, and sedimentary influences^48^. The final Bouguer anomaly, free from the surface topography effects, serves as the primary input for the inversion process, alongside seismic data, to model the Moho structure.

**Fig. 3:**
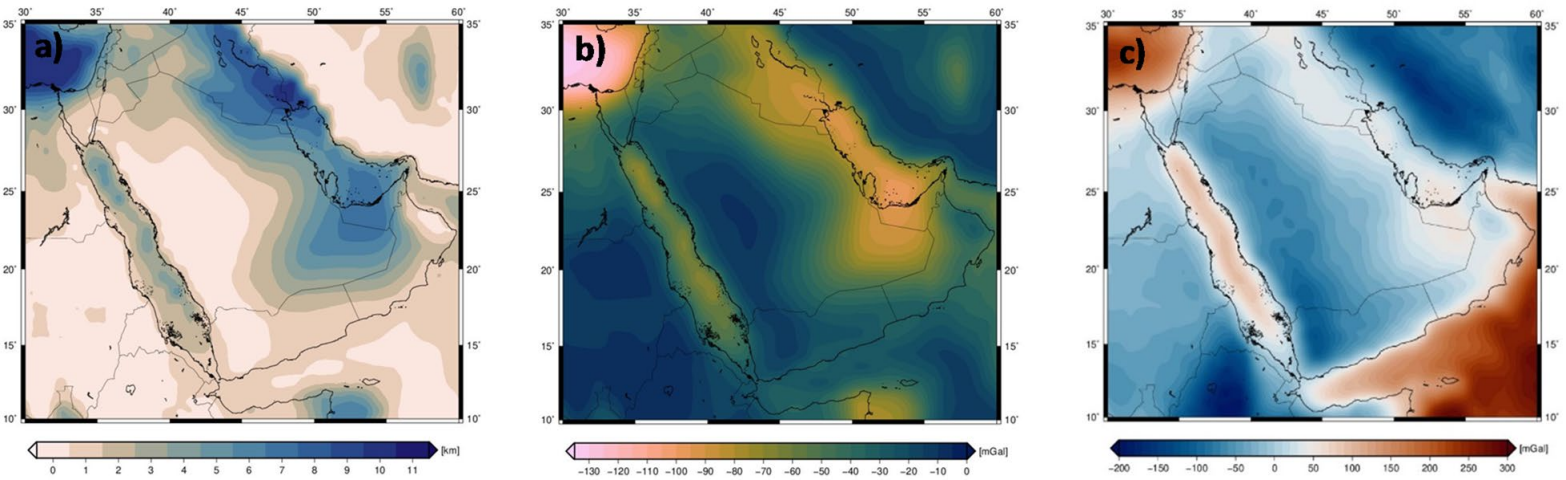
Sediment layer thickness from CRUST1.0 model (**a**), The total gravitational attraction of the sediment layer thickness (**b**), Sediment-free Bouguer anomaly obtained by subtracting the total sediment gravitational effect from the Bouguer anomaly (**c**).

Further corrections addressed the gravitational effects of sedimentary basins, as sediments typically have a lower density than the crust. These corrections, applied to the Bouguer gravity disturbance, were calculated using the CRUST1.0 model^38^, which defines three sediment layers with varying density based on depth (Fig. 3a). The gravity effect of each layer was determined by considering density contrasts relative to the underlying crust (Fig. 3b). The resulting corrected data effectively removed the influence of sedimentary layers, leaving a signal primarily attributed to the anomalous Moho relief^48^ (Fig. 3c). Access to the sediment model is available at (https://igppweb.ucsd.edu/~gabi/crust1.html).

The adjusted gravity data were utilized as input for the inversion process, integrating seismic constraints to enhance the reliability and interpretability of the methodology. The complete Bouguer gravity disturbance was calculated using the following Eqs. 4^6,47^:

1 $$\:{\:\:\:\:\:\:g}_{CBA=\:}{g}_{obs\:}-\:{g}_{t}+\left(\varDelta\:{g}_{L}+\:\varDelta\:{g}_{FA}-\:\varDelta\:{g}_{B}+\:\varDelta\:{g}_{T\:}\right)$$

Where: (g_obs_): Observed gravity at the station, $\:\varDelta\:{g}_{FA})$ : Free-air correction (gravity disturbance), $\:(\varDelta\:{g}_{L}$): Latitude correction, $\:(\varDelta\:{g}_{B})$: Bouguer correction, $\:(\varDelta\:{g}_{T\:})$: Terrain correction, and $\:\left({g}_{t\:}\right)$: Theoretical sea-level gravity (“normal gravity”).

The representation of gravity data depends on the specific adjustments applied during processing. As defined by^49^, the adjusted gravity values are obtained by subtracting normal gravity from the observed readings at each station. The equation for the complete Bouguer gravity disturbance, including sediment corrections, is expressed as:

2 $$\:{g}_{CBA\_No\_Sed}=\:{g}_{dis}-\:{g}_{TF}-\:{g}_{TC\:}-\:{g}_{B\:}-\:{g}_{MS\:}-\:{g}_{IS}\:$$

Where: $\:\left({g}_{dis}\right)$: Gravity disturbance, calculated as the observed gravity minus normal gravity adjusted for station height, $\:{(g}_{TF})$: Terrain effect correction, incorporating the actual topography and specific density variations for improved accuracy compared to the Bouguer plate approximation, $\:{(g}_{TC\:})$: Terrain constant correction, a simplified approximation of $\:{(g}_{TF})$ within a defined radius around the station, ($\:{g}_{B\:})$: Bathymetric gravity correction, accounting for the gravitational influence of water depth, ($\:{g}_{MS\:})$: Marine sediment gravity correction, addressing the mass difference between marine sediments and water, and $\:({g}_{IS}$): Inland sediment gravity correction, accounting for the mass difference between inland sediments and the underlying rock density.

The corrected gravity data, combined with seismic constraints, provided reliable input for modeling the Moho structure. By adequately accounting for all major sources of variability, the framework isolated the anomalous gravity signal attributed to the Moho relief, ensuring accuracy as the primary target of the inversion process.

### Gravity inversion

Our methodology builds upon the work of^48^ and^50^^51^,, employing a nonlinear inversion algorithm to integrate gravity and seismic data that were received from global receiver’s function and refraction investigation locations across the study area. This algorithm implemented using the Python package Fatiando (https://www.fatiando.org/), leverages the Gauss-Newton formulation of Bott’s Eq^52^.. Uieda et al. (2017)^48^ addressed the inherent instability of the Bott method by reconfiguring the ill-posed inversion problem into a well-posed framework. This was achieved using tesseroids (spherical prisms) instead of rectangular prisms and the introduction of a Tikhonov regularization parameter to stabilize the inversion process. The algorithm accounts for Earth’s curvature by using tesseroids in forward modeling. The regularization parameter plays a critical role in stabilizing the inversion by balancing the trade-off between fitting the observed data and ensuring smoothness in the model, which prevents overfitting and yields a physically plausible solution. The technique divides the Moho surface into tesseroid units. A numerical function is established to link the depth of the Moho with gravity anomalies, creating an inverse problem. This problem seeks to identify the depth parameters of each tesseroid by minimizing the discrepancy between observed and calculated gravity disturbances. At each iteration, the following nonlinear equation is solved to refine the model:

3 $$\:\left[{A}^{KT}{A}^{K}+\mu\:{R}^{T}R\right]\varDelta\:{P}^{K}={A}^{KT}\left[{g}^{o}\left({X}_{i}\right)-g\left({X}_{i},\:\rho\:,\:{P}^{K-1}\right)\right]-\mu\:{R}^{T}{R}_{{P}^{K\:}},\:i=\mathrm{1,2}\dots\:.N$$

Where: (A^K): is the Jacobian matrix at iteration K, (µ): is the regularization parameter, [(g〗^o (X_i)): is the observed gravity disturbance, (R): is an L× M finite-difference matrix representing first-order differences between adjacent tesseroids, (g(X_i,ρ,P^(K-1))): is the computed gravity disturbance at a point calculated from the depth model, (P^K): is the M-dimensional parameter vector containing Moho depths, (K): represents the iteration number, and (T): denotes the transpose operation.

A tesseroid model is designed to replicate the pre-processed gravity data and is parameterized by three key elements: (1) a regularization parameter to control the model’s smoothness (µ); (2) the reference Moho depth $\:($*Z*_*ref*_$\:)$; and (3) the density contrast at the Moho (∆ƍ). The optimal regularization parameter is determined by testing multiple subsets of the data and selecting the value that minimizes the mean square error (MSE). Cross-validation was applied to develop a reference model using predetermined values for (µ)^53^.

**Fig. 4:**
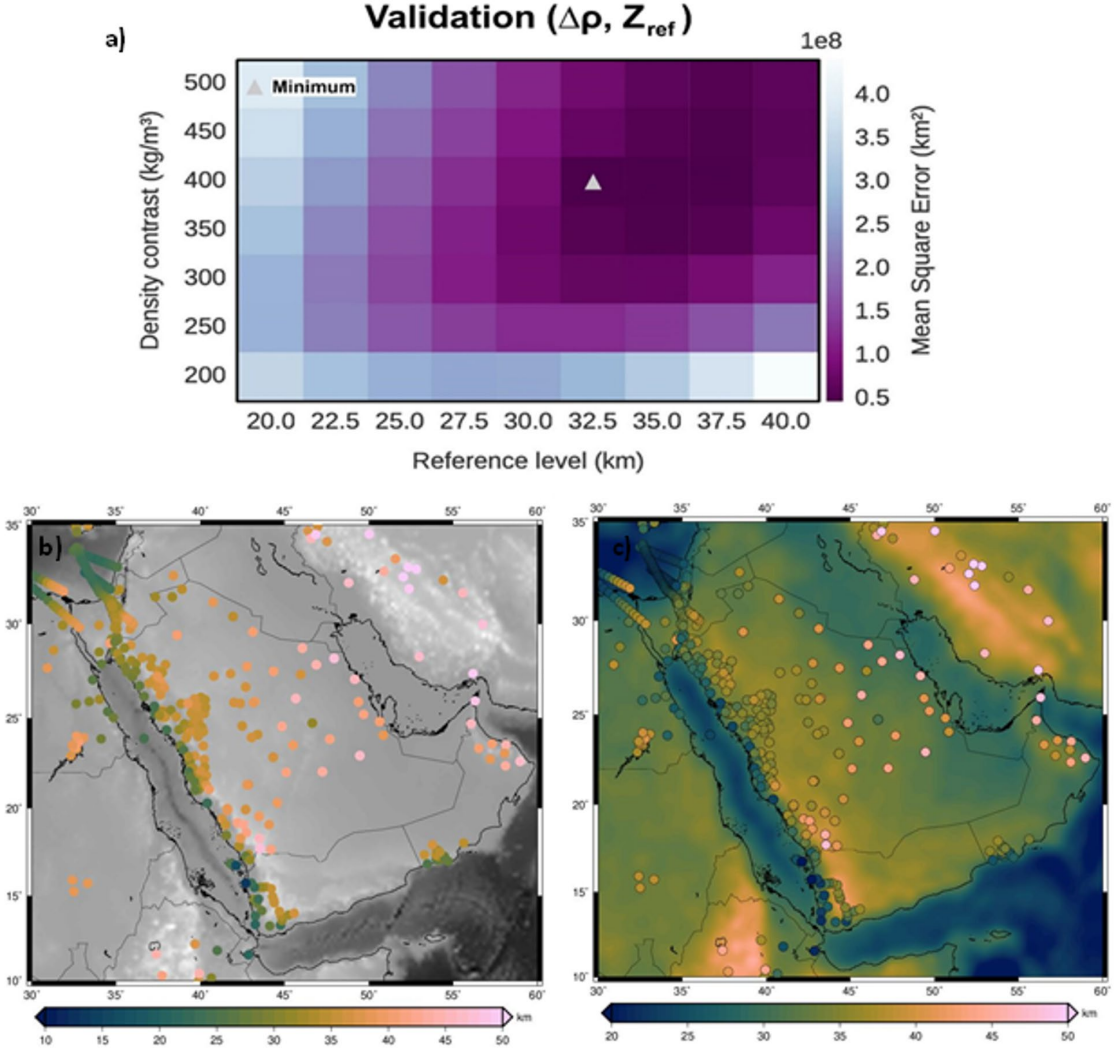
Cross-Validation results used to determine the best fit reference model with minimum MSE (white triangle) between gravimetric and seismic solutions (**a**), Seismic Moho depth estimates (**b**), Moho depth estimates from inverting the GOCE gravity data with variable density contrast (**c**).

Due to limited prior knowledge of Moho’s depth and density contrast, a broad range of values is explored. The reference depth is tested in 2.5 km increments from 20 to 40 km, while the density contrast varies in 25 kg/m³ steps between 250 and 550 kg/m³. We systematically tested different combinations of reference depth and density contrast. For each combination, we performed an inversion using a pre-selected regularization parameter (µ). The resulting models were then rigorously evaluated against seismic data with known Moho depths by calculating the Mean Squared Error (MSE). The combination that produced the lowest MSE was identified as the best-fitting model. The resulting error landscape (Fig. 4a) shows a well-defined global minimum, confirming the robustness of the selected parameters.

## Results

### Inversion gravity results

The inversion results indicate a reference Moho depth of 32.5 km and a density contrast of 400 kg/m representing the combination that provides the best fit with the data from the seismic station points as shown in Fig. 4b, as evidenced by the clear global minimum in the MSE plot (Fig. 4a). The resulting Moho depth model is shown in Fig. 4c. These values suggest a relatively uniform crust-mantle boundary across the study area, with variations in Moho topography attributed to deeper mantle processes or localized crustal features.

### Density modeling of the upper mantle

Upper mantle density heterogeneities are intrinsically linked to tectonic processes, as density contrasts generate significant stresses that influence the style and amplitude of lithospheric deformation. Understanding the density structure of the upper mantle is therefore crucial for deciphering tectonic processes and their surface expressions. In this study, we investigate density variations in the upper mantle using seismic velocities derived from the recent S-wave tomographic model of Africa, AF2019^31^.

**Fig. 5:**
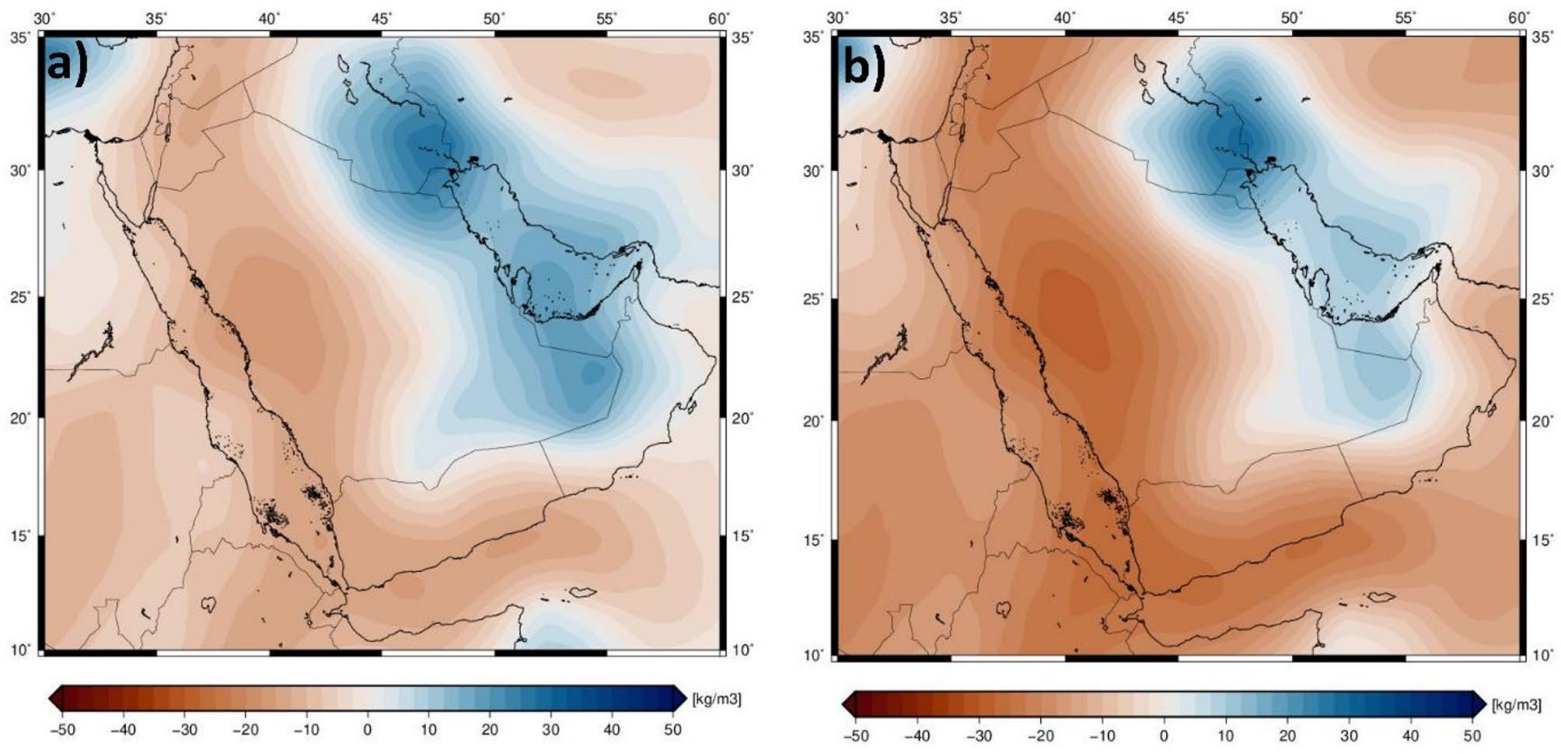
Density model of the upper mantle. Density anomalies at 100 km depth slice (reference density is 3384 kg/m^3^) (**a**), density anomalies at 150 km depth slice (reference density is 3419 kg/m^3^) (**b**).

The S-wave velocity (Vs) variations were converted to temperature and corresponding density variations using a modified version of the approach by^33^, as implemented by^54^. This conversion is based on the mineral physics approach of^55^, which allows for the derivation of density variations from seismic velocity data^55^. The resulting density model provides insights into the thermal and compositional structure of the upper mantle beneath the East ANS and its surrounding regions (Fig. 5).

### Seismic tomographic modeling

**Fig. 6:**
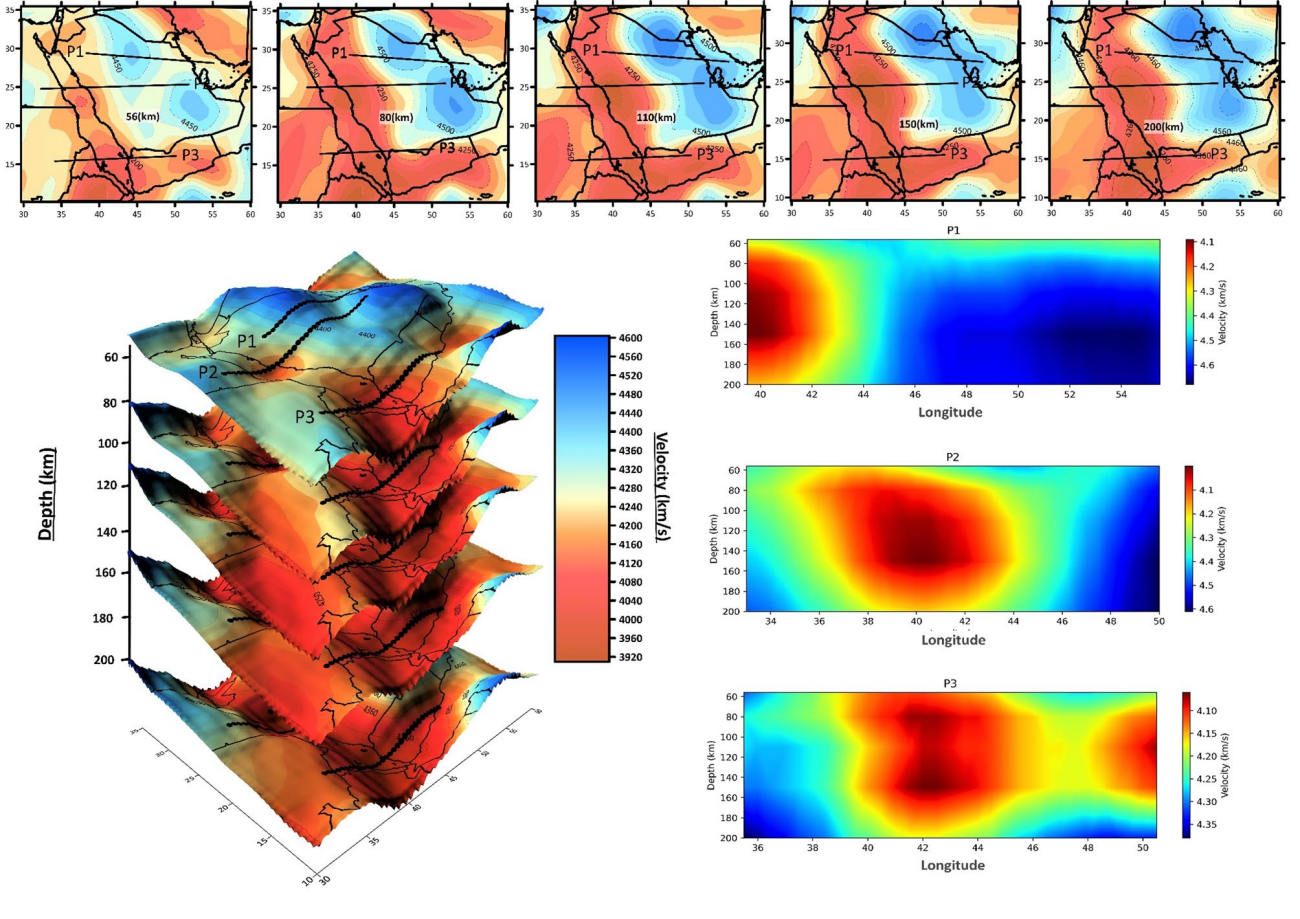
S-wave seismic velocity profiles (P1, P2, and P3) of the upper mantle density variations beneath the East ANS extracted at depths of 56 km, 80 km, 110 km, 150 km, and 200 km, reveal distinct lateral velocity anomalies indicative of thermal and compositional variations.

However, data coverage is limited and uneven due to sparse seismic stations and localized seismicity, seismic tomography is effective for studying the lithosphere and mantle, as seismic velocities reveal rock composition and temperature. In this study, we analyze S-wave seismic velocity profiles (P1, P2, and P3) to investigate upper mantle density variations beneath the East ANS and surrounding regions. The profiles, extracted at depths of 56 km, 80 km, 110 km, 150 km, and 200 km, reveal distinct lateral velocity anomalies indicative of thermal and compositional variations. The low-velocity regions (≤ 4200 km/s) correspond to thermally perturbed areas, while high-velocity regions (≥ 4600 km/s) reflect cooler, stable lithospheric blocks. These results provide insights into tectonic activity and lithospheric architecture within the region (Fig. 6).

The cross-sectional seismic velocity profiles (P1, P2, and P3) provide detailed insights into the vertical and lateral variations of the upper mantle beneath the East ANS. The Arabian Shield to Arabian Platform transition (P1) shows a broad low-velocity region (≤ 4200 km/s) at depths between 100 km and 150 km, marking the transition between these tectonic units. The high-velocity anomaly beneath the Arabian Platform extends deeper (> 200 km), indicating a thick, thermally stable lithosphere. These results suggest that the Arabian Shield is undergoing thermal perturbation, while the Arabian Platform remains relatively stable. The deeper high-velocity anomaly beneath the Platform confirms its cratonic nature, consistent with Precambrian lithospheric roots observed in global cratons^56^ (Fig. 6).

As for the Red Sea region (P2), a significant low-velocity anomaly extends from shallow depths (~ 60 km) to deeper levels (~ 200 km), with shallow-to-deep low-velocity anomaly (~ 3920 km/s). This anomaly suggests mantle upwelling and lithospheric thinning, consistent with active rifting in the Red Sea region. On either side of the anomaly, high-velocity zones are observed, indicating a relatively stable lithospheric structure. Similar velocity reductions have been documented beneath the East African Rift^57^, supporting our interpretation of Red Sea rifting dynamics (Fig. 6).

In the Nubian Shield region (P3), the cross-section reveals significant velocity variations, highlighting a complex mantle structure. Low-velocity anomalies are observed between 60 km and 150 km depths, likely associated with localized mantle upwelling and partial melting. At greater depths (200 km), high-velocity regions (> 4500 km/s) indicate the presence of older, rigid lithospheric material. This heterogeneity mirrors findings in the Saharan Metacraton, where localized upwelling interacts with ancient lithosphere^58^ (Fig. 6).

### Effective elastic thickness (EET)

**Fig. 7:**
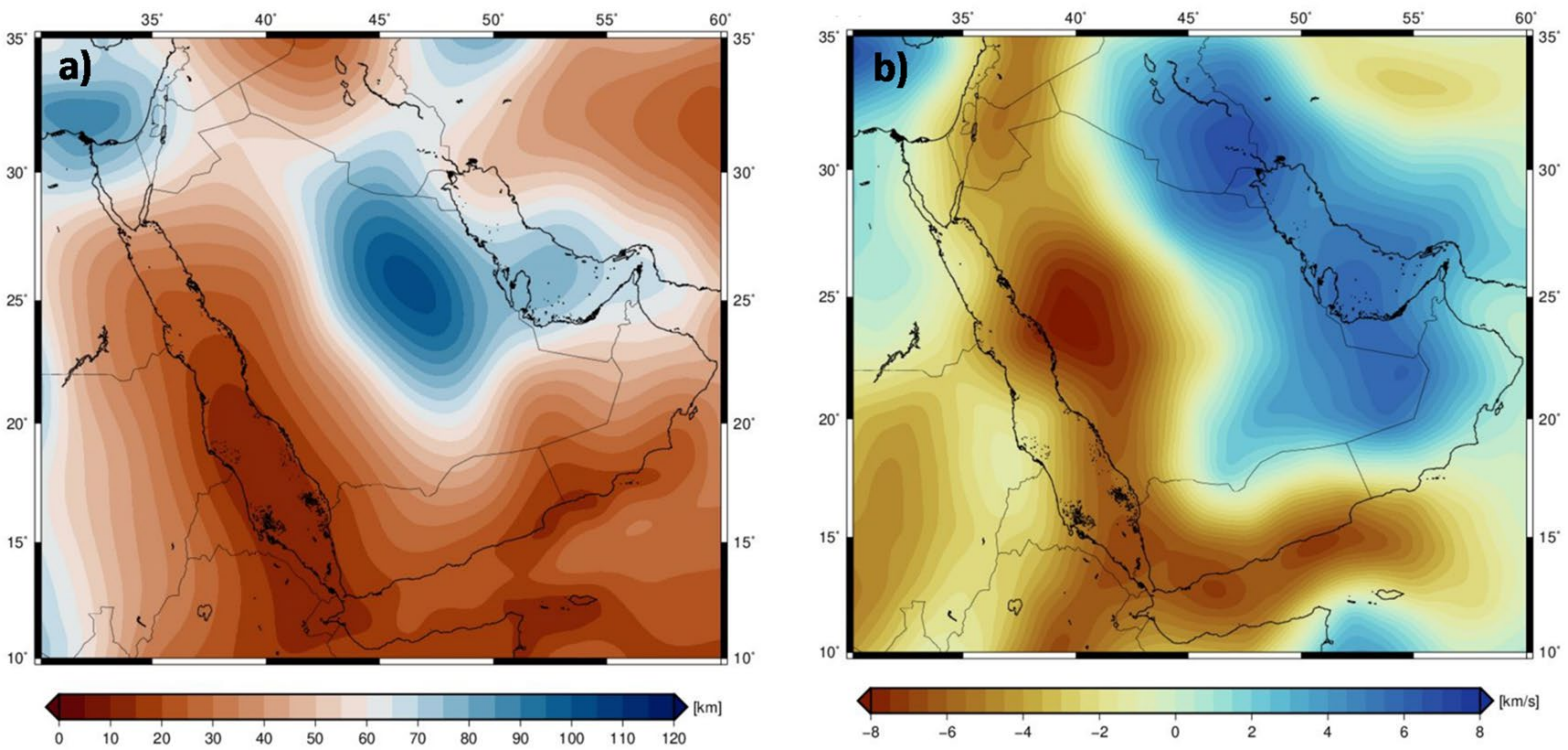
The effective elastic thickness of the ANS and surroundings **(a**), S-wave tomography model AF2019 [23] at depth slice 100 km (**b**).

The effective elastic thickness (EET) of the lithosphere is defined as the thickness of a uniform elastic plate that would respond to applied loads in the same way as the naturally heterogeneous lithosphere. As the EET is primarily controlled by the lithosphere’s thermal state and composition, it serves as a key parameter for assessing its mechanical strength and thermal variations. In this study, the EET was determined using the coherence analysis method between Bouguer gravity anomalies and topography data. This approach is based on the principle that the correlation (coherence) between the gravity field and topography is a direct function of the lithosphere’s flexural rigidity. We specifically employed a wavelet-based admittance and coherence analysis^59^, which allows for a robust, high-resolution joint analysis of the gravity field, topography, and sedimentary basin structure. The EET value at each location was estimated by fitting the observed coherence function to a theoretical model of an elastic plate in isostatic equilibrium. This method provides an independent measure of the integrated lithospheric strength, which is crucial for interpreting the thermal and density structures derived from other geophysical data. The analysis of EET reveals significant spatial variations in lithospheric strength across the study area. Thick EET values (50–75 km) are mapped along the Arabian Platform, consistent with the properties of old, cold, and tectonically stable continental lithosphere. In contrast, thin EET values (< 30 km) and weak lithosphere characterize the Red Sea and Gulf of Aden, correlating with zones of active rifting and elevated thermal gradients. These regions coincide with low S-wave velocities in the upper mantle, as resolved by the seismic tomography model of^31^. The tomography further highlights a stark contrast between the Arabian Platform (high-velocity, ~ 4.5–4.8 km/s) and the Arabian Shield, Red Sea, and Gulf of Aden (low-velocity, ≤ 4.2 km/s). These velocity anomalies are corroborated by negative density contrasts, high subsurface temperatures, and reduced lithospheric thickness (≤ 80 km) beneath the rift zones (Fig. 7a).

**Fig. 8:**
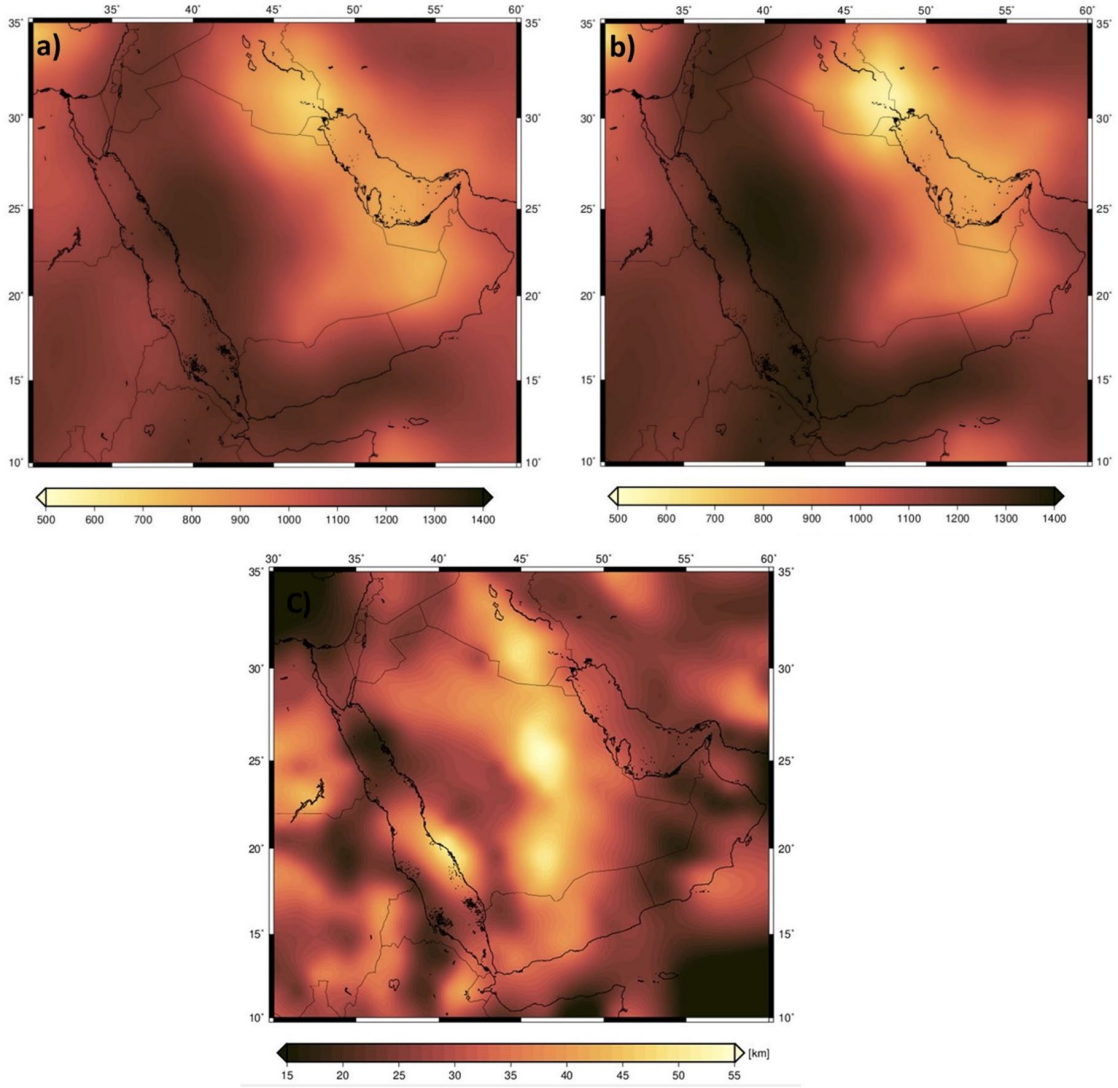
3D thermal variations model at 100 km depth slice (**a**), 150 km depth slice (**b**), and the CPD estimations from satellite magnetic data inversion (**c**).

The subsurface temperature distribution derived from S-wave tomography (Fig. 7b). reveals a clear dichotomy in mantle structure across the East ANS. The low-temperature anomaly beneath the Arabian Platform (extending to ~ 200 km depth) corresponds to high S-wave velocities (≥ 4200 km/s), indicative of a thick, stable lithosphere. This is consistent with the AF2019 tomographic model^31^, which identifies high-velocity anomalies beneath cratonic blocks like the Arabian Platform. In contrast, the high-temperature anomalies beneath the Red Sea, Gulf of Aden, and Arabian Shield correlate with low S-wave velocities (≤ 3920–4200 km/s), reflecting a thermally perturbed upper mantle and lithospheric thinning (Fig. 8a, b). These observations align with studies documenting reduced velocities in regions active mantle upwelling^31,60^. The spatial distribution of the CPD estimations from satellite magnetic data inversion, reveals significant and systematic variations across the ANS (Fig. 8c). The shallowest CPD values (**<** 25 km) form a pronounced, continuous band along the entire Red Sea rift, extending into the western and southern margins of the Arabian Shield. In stark contrast, the deepest CPD values (**>** 40 km) are confined to the northeastern Arabian Platform. A clear transitional gradient is observed, with CPD depths increasing progressively from the Red Sea coast (**~** 20–25 km) towards the stable continental interior of the Platform (**~** 35–40 km). This coherence between the crustal CPD and mantle-derived parameters robustly reinforces the identified geothermal anomalies (Fig. 8c).

### The LAB and heat flow model

**Fig. 9:**
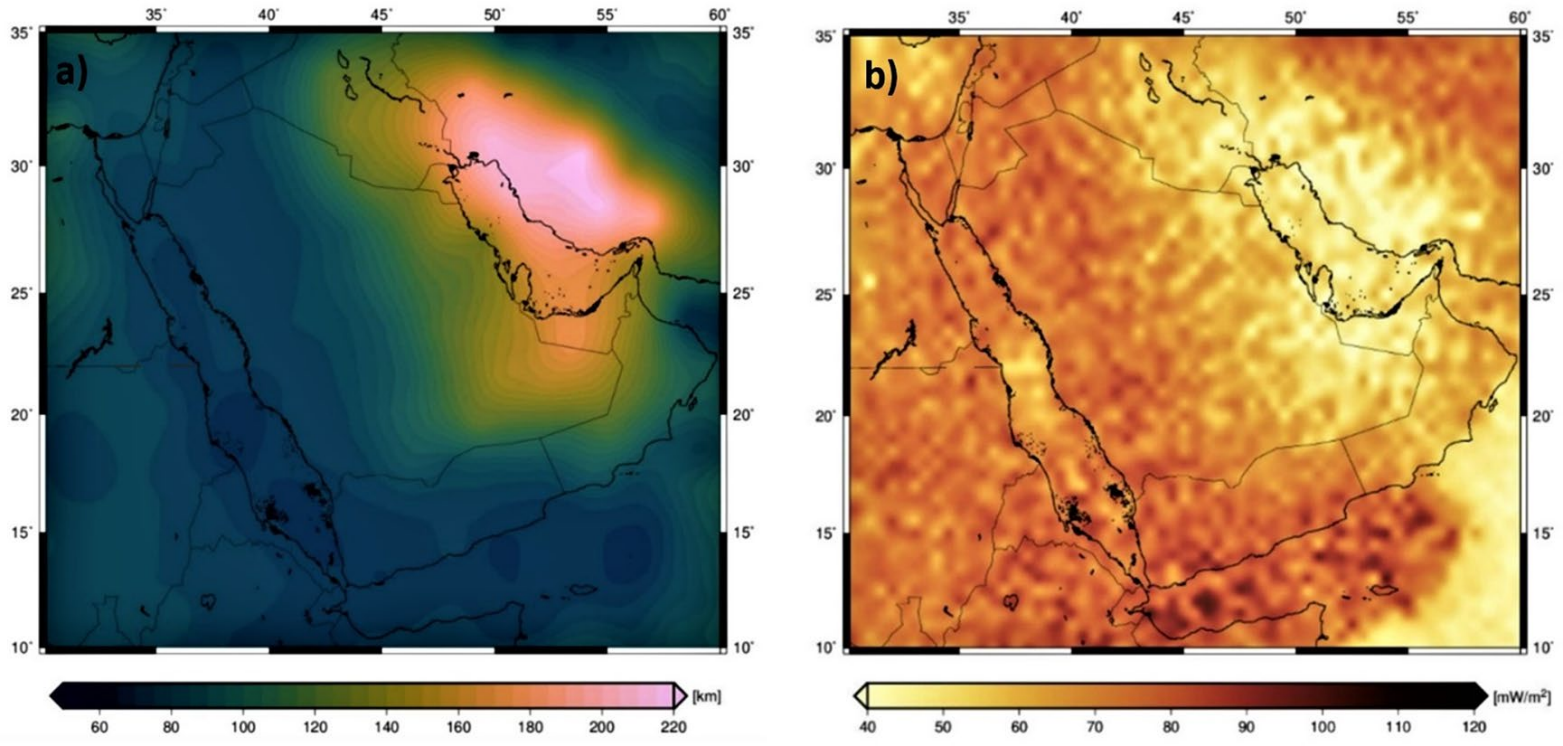
Modelled Lithosphere-Asthenosphere Boundary (LAB) (**a**), and the modeled surface heat flow (**b**).

The modeled Lithosphere-Asthenosphere Boundary (LAB) depth map reveals considerable spatial variations across the East ANS and adjacent regions. The shallowest LAB depths, ranging from approximately 60 to 120 km, are observed beneath the Arabian Shield and the Red Sea. The Red Sea exhibits the thinnest lithosphere, with LAB depths between 60 and 80 km, consistent with its active rift setting. Similarly, the Arabian Shield, despite its cratonic origins, shows moderately thin lithospheric thicknesses of 80 to 120 km, suggesting thermal modifications over time. In contrast, the deepest LAB depths, exceeding 200 km, are identified beneath the Arabian Platform and the northeastern Arabian Peninsula, characterized by a thick, stable lithosphere and high S-wave seismic velocities (4.5–4.8 km/s). Between these two extremes, transitional LAB depths of 120 to 160 km mark the boundary between the Arabian Shield and the Arabian Platform, coinciding with lateral seismic velocity heterogeneities observed in mantle cross-sections and reflecting variations in lithospheric composition and thermal evolution (Fig. 9a).

The modeled surface heat flow map of the ANS and surrounding regions reveals distinct spatial variations in geothermal heat flux, highlighting the thermal structure of the lithosphere. The highest heat flow values (≥ 100 mW/m²) are concentrated in the Red Sea and Gulf of Aden, with peak values exceeding 120 mW/m², corresponding to actively extending lithosphere and mantle upwelling. In contrast, the Arabian Shield exhibits moderate heat flow (70–100 mW/m²), reflecting a lithosphere that has undergone thermal modification. The lowest heat flow values (< 70 mW/m²) are found beneath the Arabian Platform, consistent with the presence of thick, stable lithospheric roots that insulate the surface from deeper mantle heat sources. (Fig. 9b).

### MODIS-derived land surface temperature (LST)

**Fig. 10:**
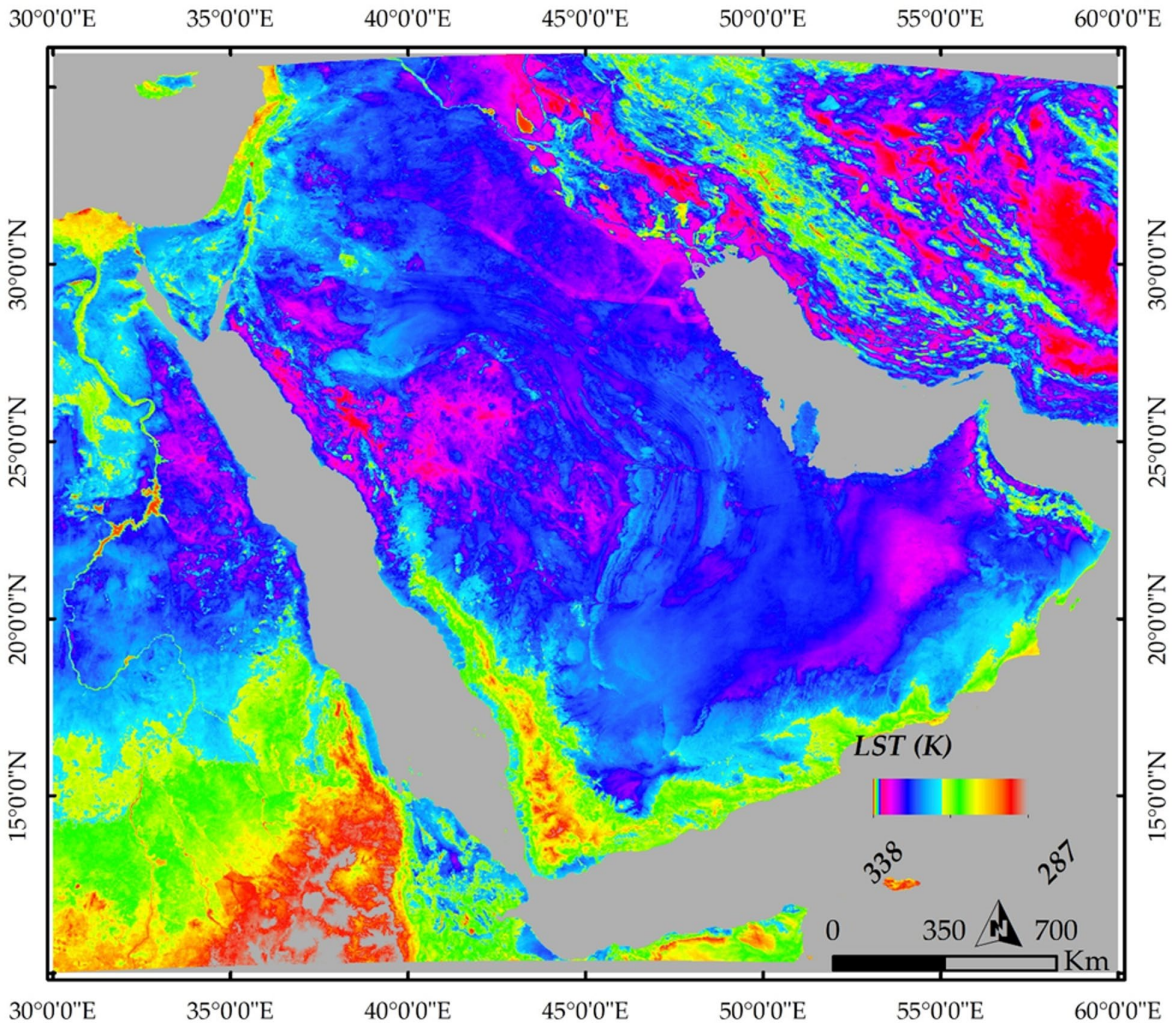
MODIS-derived LST map of the study area, clearly shows higher surface temperatures (pinkish hues) over the Arabian Shield and Nubian Shield, contrasting with the cooler Arabian Platform (blue), consistent with geophysical findings of a thinner, more thermally active lithosphere in the shields and a thick, stable one under the platform.

In addition to comprehensive geophysical investigations that clearly delineated the subsurface conditions using multiple datasets. The current study also integrated MODIS LST data to spatially map thermal patterns across the entire study area (Fig. 10), using averaged data over an eight-year period. Surface thermal anomalies often serve as preliminary indicators of complex subsurface features, especially in regions with limited direct access. MODIS LST products have been extensively used across various disciplines, including geothermal studies. For example, Li et al. (2012)^61^ employed MODIS LST imagery to detect geothermal anomalies in Tengchong, Yunnan Province, China, with findings that aligned well with geothermal gradient measurements.

Our results demonstrate a high degree of consistency between the LST patterns and the findings from geophysical datasets. Notably, the ANS exhibits clear heterogeneity in surface temperatures, with elevated LST values over the Arabian Shield and Nubian Shield compared to the adjacent Arabian Platform. This is visually apparent on the LST map (Fig. 10), where the shields are highlighted in distinct pinkish hues, indicating relatively higher surface temperatures. The Arabian Shield, in particular, shows more intense thermal anomalies than the Nubian Shield, which aligns well with our geophysical depth analyses (Table 2).

LST variations are consistent with differences in the deeper lithosphere, as revealed by geophysical data analysis. The Arabian Platform is characterized by a thick, stable, and thermally cool lithosphere, associated with low surface heat flow, reflecting its cratonic nature. In contrast, the Arabian Shield and the Red Sea region show a thinner, more thermally active lithosphere. It is worth noting that the pinkish zones highlighted in the LST map coincide with areas known for significant mineral alteration, volcanic activity, fault systems, and magnetic anomalies—features that are well-documented indicators of geothermal potential^63^.

### Discussion

Our integrated multi-parametric analysis reveals a fundamental tectonic dichotomy within the Arabian-Nubian Shield (ANS), delineating a clear geothermal architecture with direct implications for exploration. The convergence of satellite gravity, seismic tomography, thermal modeling, and surface data consistently identifies three distinct geothermal provinces. The high-enthalpy Red Sea rift system is characterized by a thin lithosphere (60–80 km), elevated heat flow (> 100 mW/m²), shallow Curie Point Depth (< 25 km), and low S-wave velocities (≤ 3920 m/s), all indicative of active mantle upwelling. This configuration shares direct similarities with the East African Rift System, where analogous processes sustain world-class geothermal fields. In contrast, the moderate-enthalpy Arabian Shield exhibits a thinner lithosphere (80–120 km) and higher heat flow (70–90 mW/m²) than the low-enthalpy Arabian Platform, which retains a thick cratonic root (> 200 km) and low heat flow (40–50 mW/m²). This systematic classification, validated against previous seismic and geophysical studies (Table 2), provides a robust, multi-parameter framework for prioritizing exploration.

The consistent spatial correlation between deep lithospheric structure and surface manifestations enables a strategic shift from speculative to knowledge-driven geothermal exploration. For energy policymakers, our study provides the scientific foundation to designate the Red Sea coastal zone and western Arabian Shield as strategic geothermal development corridors and to establish differentiated incentive structures that reflect the varying resource temperatures across these provinces. For exploration companies, our integrated approach suggests a cost-effective, phased strategy: initial regional screening using the geophysical parameters defined here (e.g., LAB depth, heat flow, CPD), followed by target refinement via focused geophysical surveys, and culminating in exploration drilling prioritized where multiple parameters indicate favorable conditions for heat source and reservoir development. The strong correlation between MODIS Land Surface Temperature anomalies and subsurface thermal parameters offers a particularly valuable low-cost tool for preliminary screening.

By adopting this geophysically-guided framework, stakeholders can significantly reduce exploration risk and accelerate the development of the ANS’s substantial geothermal resource. The Red Sea province offers potential for conventional high-temperature development, while the Arabian Shield may be suitable for both conventional medium-temperature applications and enhanced geothermal systems, akin to the Basin and Range Province in the western United States. This approach paves the way for harnessing a clean, baseload energy source, thereby supporting regional energy security and contributing to global climate goals.

**Table 2:** Comparison of geothermal and lithospheric characteristics across regions of the ANS.

Region		Recent Study – Key Results	Previous Studies – Matching Findings
Arabian Platform	Moho Depth	Moho ~ 32.5 km	^23^– Moho > 30 km^31^
	LAB Depth	LAB > 200 km	^31^– LAB > 200 km
	Heat Flow	Heat flow ~ 40–50 mW/m²	^29^– ~50 mW/m²
	Seismic Velocity	S-wave velocity ≥ 4200 m/s	^26^– ≥4200 m/s under Platform
Arabian Shield	Moho Depth	Moho 25–30 km	^24^– Moho 22–25 km
	LAB Depth	LAB 80–120 km	^24^– LAB 100–110 km
	Heat Flow	Heat flow ~ 70–90 mW/m²	^28^– 45–60 mW/m²
	Seismic Velocity	S-wave velocity ≤ 4200 m/s	^26^– ≤4200 m/s
Red Sea	LAB Depth	LAB 60–80 km	^31^– LAB < 80 km
	Heat Flow	Heat flow ≥ 100 mW/m²	^29^– >100 mW/m²
	Seismic Velocity	S-wave velocity ≤ 3920 m/s	^30^– <3920 m/s
	EET	Thin EET (< 30 km)	^59^– Thin lithosphere in rift
Nubian Shield	Shallow Mantle	Low-velocity anomalies at 60–150 km	^58^– 60–150 km low velocity
	Deep Mantle	High velocities at ~ 200 km	^58^– >200 km high velocity
	Thermal Pattern	Mixed hot/cold zones, partial melting	^62^– Heterogeneous mantle

## Conclusion

This study demonstrates that the geothermal potential of the ANS is intricately linked to its upper mantle structure, as revealed by S-wave tomography, seismic velocity profiles, and density modeling. The observed heterogeneities in density—characterized by a stable, high-density lithosphere beneath the Arabian Platform and a thermally perturbed, low-density mantle beneath rifting zones—support seismic tomographic studies and underscore the dynamic tectonic evolution of the ANS. These findings highlight that future geothermal exploration should prioritize regions with lithospheric thinning and mantle upwelling, where elevated heat flow and permeable fault systems could enhance resource viability.

The study also emphasizes the value of integrating geophysical and geological tools in geothermal modeling, an approach that can be applied to other tectonically complex regions. By correlating temperature heterogeneities, density variations, and seismic velocity anomalies, it is clear that the ANS’s geothermal potential is governed by the dynamics of its upper mantle. The stable, low-temperature, high-velocity Arabian Platform contrasts with the active, high-temperature, low-velocity regions beneath the Red Sea and Arabian Shield, reflecting ongoing mantle processes. These findings align with regional and global studies, providing a solid foundation for future geothermal exploration.

Furthermore, the study highlights the heterogeneous lithospheric structure of the East ANS, shaped by ongoing rifting, cratonic stabilization, and mantle-driven thermal processes. The integration of LAB depth modeling with seismic tomography offers valuable insights into the region’s geothermal and tectonic evolution. The differences between the actively deforming Red Sea rift system, the thermally modified Arabian Shield, and the tectonically stable Arabian Platform are particularly noteworthy. Future research incorporating gravity and magnetotelluric data could refine our understanding of melt distribution, lithospheric composition, and deeper mantle interactions, ultimately providing a more comprehensive view of the lithosphere-asthenosphere system in this geodynamically complex region.

Finally, the surface heat flow distribution across the ANS and adjacent regions reveals a strong correlation with lithospheric thickness, rift dynamics, and mantle thermal processes. The highest heat flow values are observed in active rifting zones (Red Sea, Gulf of Aden), while the lowest are found beneath the stable Arabian Platform. These insights contribute to a deeper understanding of lithospheric thermal evolution and have significant implications for geothermal resource potential in the region.

## Data Availability

Data are available from the corresponding author upon reasonable request.

## Code Availability

Code used for the analysis is available upon reasonable request.
